# Use of the gonadal vein for kidney transplantation in patients with iliac venous complications: case series

**DOI:** 10.1186/s12882-025-04510-8

**Published:** 2025-10-22

**Authors:** Sammara Azevedo Guedes, Gabriel Brayan Gutiérrez-Peredo, Rodrigo Mota, Thiago Abud Menezes, Fernanda Pinheiro Martin Tapioca, Pedro Botelho Alencar, Milena Sampaio Barreto Machado, Camila Rodrigues Durando, Victoria Andrade Lobo, Ricardo José Costa Mattoso

**Affiliations:** 1 Ana Nery Hospital, Federal University of Bahia, R. Saldanha Marinho, s/n - Caixa D’água, Salvador, BA 40301-155 Brazil; 2 Department of Nephrology and Kidney Transplantation, Ana Nery Hospital, Federal University of Bahia, Salvador, BA Brazil; 3 Doctoral and Master Postgraduate Program in Medicine and Health, Federal University of Bahia, Salvador, BA Brazil; 4 University of Salvador - UNIFACS, Salvador, BA Brazil; 5 Bahiana School of Medicine and Public Health, Salvador, BA Brazil; 6 Faculty of Medicine, Major University of San Simón, Cercado, CBBA Bolivia

**Keywords:** Gonadal vein, Venous complications, Iliac thrombosis, Iliac artery stenosis, Kidney transplantation

## Abstract

This study presents four urgent deceased-donor kidney transplants in women with vascular access failure, where the gonadal vein was used for venous anastomosis. Two patients were high immunological risk. Cold ischaemia time ranged from 13h47min to 26h20min. Ureteral anastomoses used Lich-Gregoir or uretero-ureteral techniques. Complications included wound infection, pyelonephritis, and antibody-mediated rejection; no vascular issues or reoperations occurred. Hospital stays ranged from 7 to 39 days. Renal function improved, stabilizing below 1.5 mg/dL. Gonadal vein anastomosis appears to be a feasible and safe alternative in complex transplant scenarios.

## Clinical trial number

Not applicable.

## Introduction

Kidney transplantation remains the best treatment for end-stage chronic kidney disease (CKD) [1–5]. However, patients with long-standing CKD and prolonged dialysis may develop iliac vein complications, such as thrombosis or calcification, making conventional venous access unfeasible [6, 7]. In such cases, the gonadal vein may offer a viable alternative for venous anastomosis [8, 9]. This study describes four cases using this technique and assesses their surgical as well as clinical outcomes.

## Methods

This retrospective study describes four patients who underwent emergency kidney transplantation due to vascular access failure at Hospital Ana Nery (2013–2025) [10, 11]. The causes of vascular insufficiency were identified by angiotomography. All patients received induction immunosuppression. We describe the clinical, laboratory, and surgical outcomes after 1 year of follow-up (Tables 1 and 2).

**Table 1:** Characteristics of kidney transplant patients with gonadal vein anastomosis

Variables	CASE 1	CASE 2	CASE 3	CASE 4
**Sociodemographic variables**				
Age, years	40	45	27	59
Sex	Female	Female	Female	Female
Race	Black	Mixed-Race	Mixed-Race	Mixed-Race
Months on hemodialysis	276	180	120	366
BMI	31.6	22.4	18.6	22.8
**Clinical and surgical data**				
High risk	No	Yes	No	Yes
Expanded criteria	No	No	No	No
Induction therapy (Thymoglobulin)	Yes	Yes	*Yes*	Yes
Donor-specific antibodies	No	Yes	No	No
**Immunosuppression therapy**				
Group 1 (PND + FK + AZA)	No	No	Yes	No
Group 2 (PDN + FK + MPS)	Yes	Yes	No	Yes
Immediate Graft Function	No	No	Yes	Yes
Cause of Chronic Kidney Disease	SLE	Chronic glomerulonephritis	Indeterminate	Indeterminate
Vascular Access Failure	Yes	Yes	Yes	Yes
**Creatinine level**,** mg/dL**				
Creatinine, post-transplant	8.4	5.0	11.0	10.5
Creatinine, 7–10 days after	6.4	1.7	6.3	1.2
Creatinine, one month after	0.9	2.0	1.5	1.1
Creatinine, three months after	1.2	0.7	1.7	1.1
Creatinine, six months after	0.9	0.8	1.3	1.2
Creatinine, one year after	1.2	0.8	1.3	1.2
Kidney function - CKD-EPI	49.0	62.4	63.2	52.1

**BMI =** Body Mass Index; **PND =** Prednisone; **FK =** Tacrolimus; **AZA =** Azathioprine; **MPS =** Mycophenolate Sodium; **CKD-EPI =** Chronic Kidney Disease Epidemiology Collaboration; **SLE =** Systemic Lupus Erythematosus; **eGFR =** Estimated glomerular filtration rate

**Table 2:** Main surgical features and donor characteristics

Variables	CASE 1	CASE 2	CASE 3	CASE 4
**Surgical**				
Cold ischemia time, hours	13:47	16:15	23:50	26:20
Warm ischemia time, minutes	63	70	38	50
Number of HD after transplantation	4	3	2	0
Arterial anastomosis	Common iliac artery	Infra-renal aorta	Common iliac artery	Infra-renal aorta (orthotopic)
Venous anastomosis	Gonadal vein	Gonadal vein	Gonadal vein	Gonadal vein
Ureteral implantation, technique	Lich-Grégoir	Uretero-ureteral	Uretero-ureteral	Uretero-ureteral
Resistance index, post-transplantation	< 0.7	0.52	0.6	0.76
Postoperative infections	Surgical wound	No	Histological pyelonephritis /AMR	Pyelonephritis
Vascular complication	No	No	No	No
Reoperation	No	No	No	No
Hospitalization time, days	39	8	11	7
**Donor characteristics**				
Donor class	Death	Death	Death	Death
Types of brain death	Skull trauma	Underground*	Skull trauma	Skull trauma
Age, years	36	9	35	27
Sex	Male	Male	Male	Male
Race	Mixed-Race	Mixed-Race	Mixed-Race	Black
BMI	26.3	15.31	25.9	22.9
Input creatinine	0.9	1.3	1,4	1.1
Final creatinine	2.3	1.0	0.6	0,9
Last creatinine ≥ 1.5	Yes	No	No	No
Diuresis in 24 h (mL)	2650	1170	800	1300

**HD = **hemodialysis;** BMI =** Body mass index; **AMR =** Antibody-mediated acute rejection

* Refers to death caused by asphyxia secondary to a landslide, in which the patient was buried under soil and debris

### Surgical technique

**Fig. 1:**
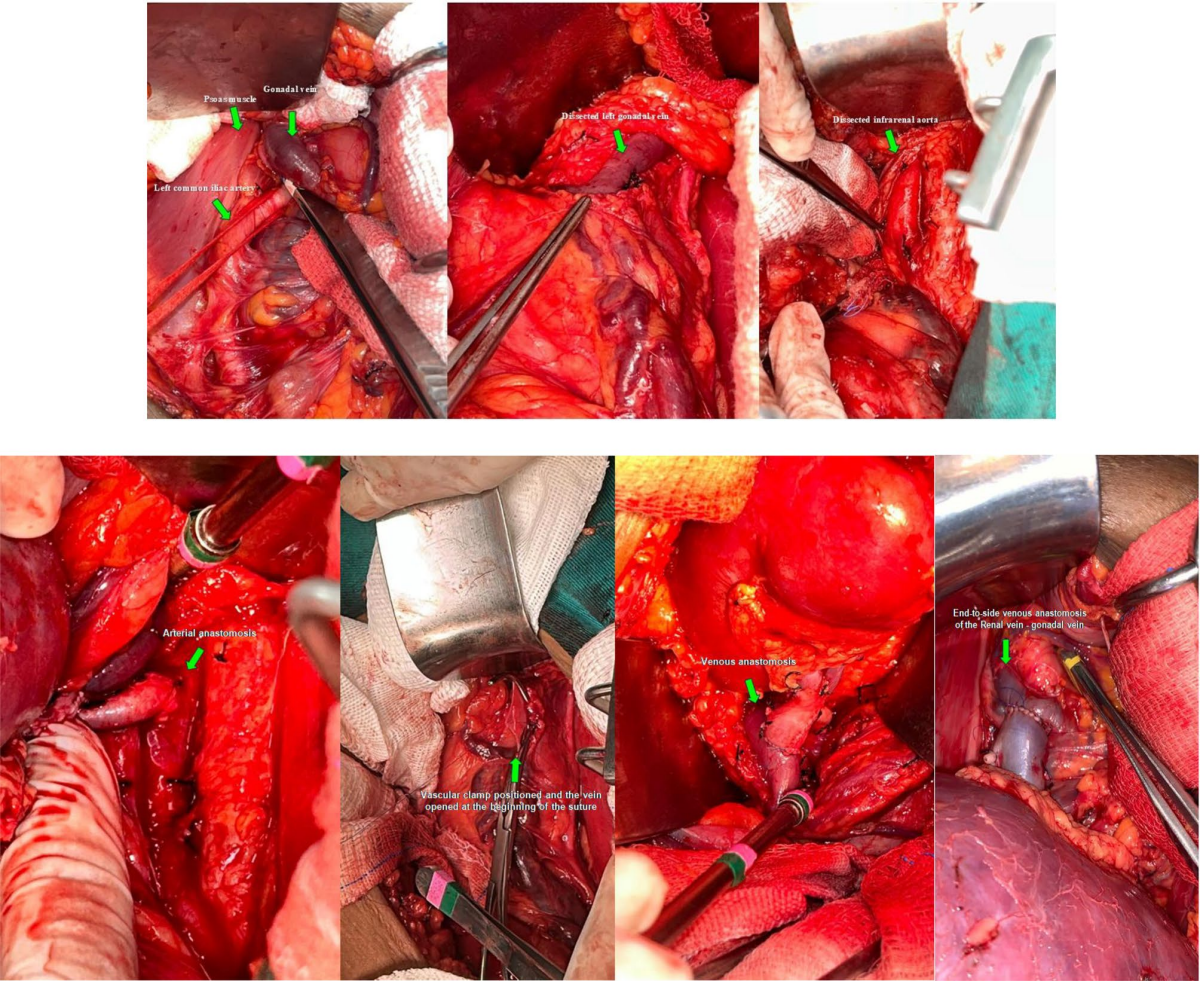
**a-g.** (**a**) Exposure of the left common iliac artery, psoas muscle, and gonadal vein. (**b**) Identification and dissection of the left gonadal vein. (**c**) Dissection of the infrarenal aorta. (**d**) Vascular clamp in place and vein opened at the start of the suture. (**e**) Arterial anastomosis. (**f-g**). End-to-side venous anastomosis of the renal vein and gonadal vein. *Images courtesy of Dr. Rodrigo Mota*

**Fig. 2:**
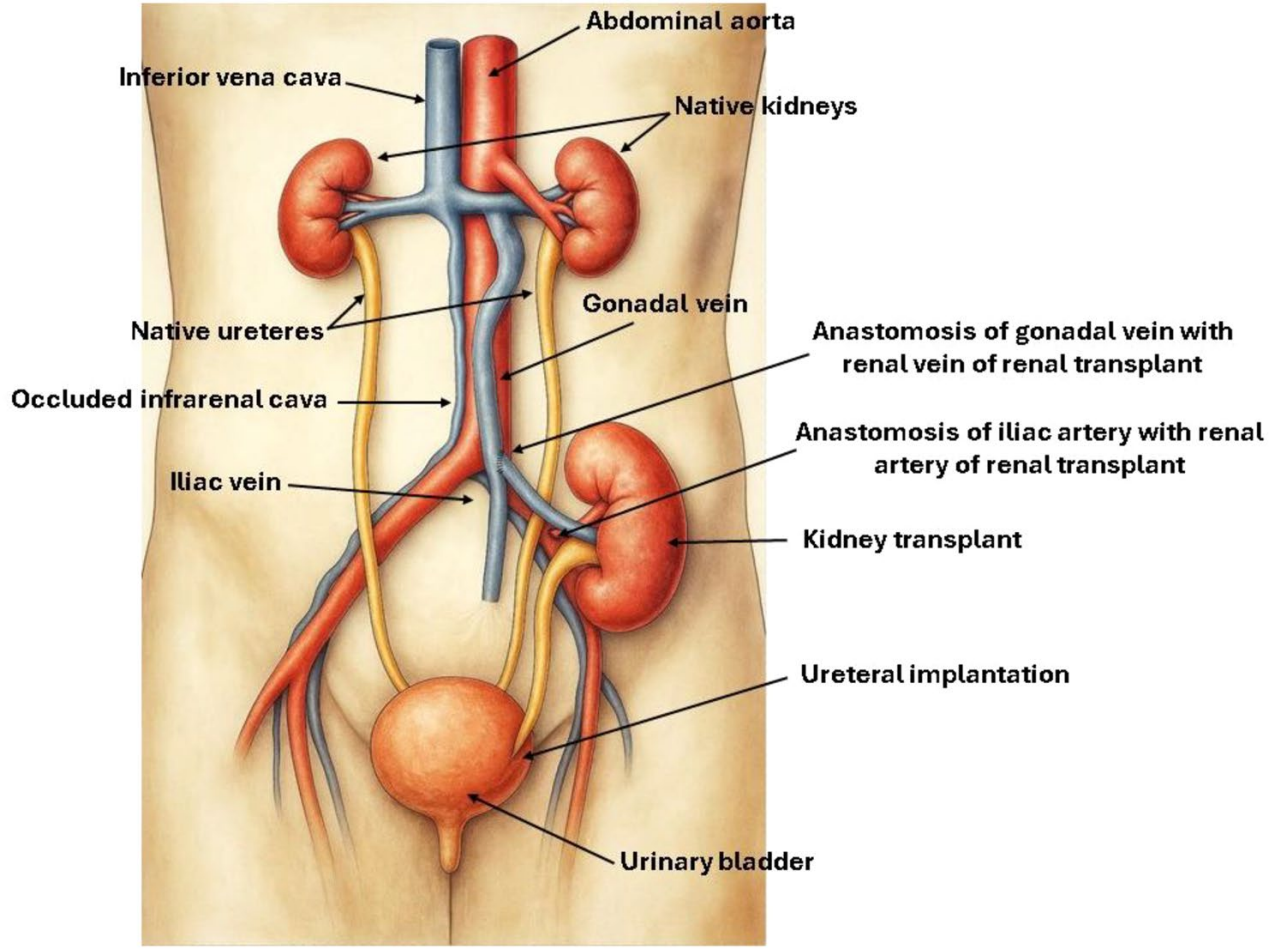
Line drawing. The figure was initially designed by *Victoria Andrade Lobo*, with subsequent graphic editing and refinement performed by *Yren Montaño Balderrama* and* Iris Montaño-Castellón*

The procedure begins with a pararectal incision—preferably on the left side, although the right may also be used—followed by extraperitoneal access. The gonadal vein, serving as a vicariant conduit, is identified and a short segment is carefully dissected to minimize the risk of injury to adjacent lymphatic structures or collateral varicosities. Dissection continues meticulously along the course of the vein, which lies in close proximity to the ureter, ensuring that surrounding anatomical structures remain undisturbed. Arterial dissection is subsequently undertaken, with the common iliac artery being the preferred site for arterial anastomosis. In one of the cases described below, however, the infrarenal aorta was utilized. Vascular anastomosis is performed using an end-to-side technique (Figs. 1a-g and 2).

### Case 1

**Fig. 3:**
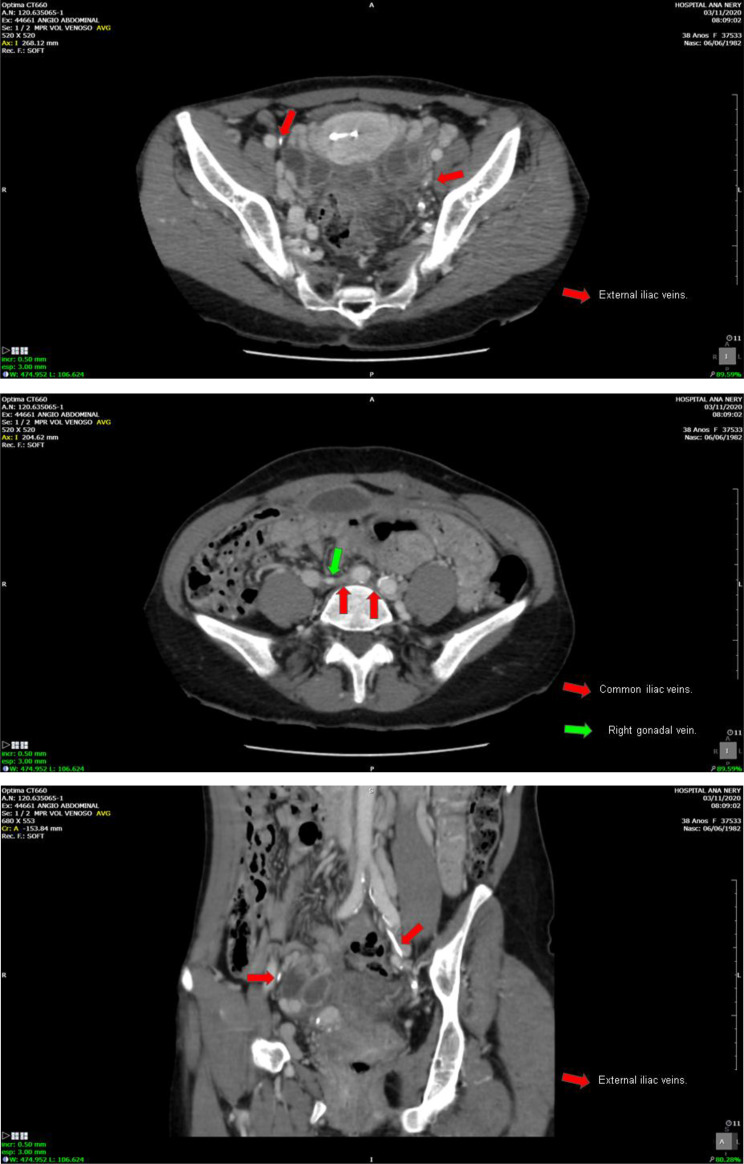
**a-c**. Common iliac veins with signs of chronic occlusion. Right external iliac vein with signs of chronic occlusion and luminal calcification. Left external iliac vein with signs of partial chronic thrombosis and luminal calcification. *Images courtesy of Dr. Thiago Abud Menezes*

A 40-year-old female patient underwent hemodialysis for 276 months. CKD was secondary to Systemic Lupus Erythematosus and Lupus Nephritis. She presented vascular failure and chronic occlusion and luminal calcification in the common iliac veins, which made conventional venous access unfeasible for this patient (Fig. 3a-c). The cold ischemia time (CIT) in this case was 13 h 47 min. A termino-lateral anastomosis of the renal vein to the gonadal vein was performed, and the termino-lateral anastomosis of the renal artery was done to the common iliac artery. The surgical technique used for the ureteral implant was through the uretero-vesical anastomosis, using the Lich-Greior technique, with the implantation of a double-J ureteral stent.

The immunological risk was low, and she received thymoglobulin as induction therapy. For maintenance immunosuppression, prednisone (PDN), tacrolimus (FK), and mycophenolate sodium (MPS) were used. Graft function was not immediate, with an initial creatinine of 8.4 mg/dL, which decreased and stabilized at 1.2 mg/dL after one year. The patient did not require any further surgical interventions. However, she developed a wound infection caused by *Escherichia Coli* and *Klebsiella Pneumoniae* producing Extended Spectrum Beta-Lactamase (ESBL). The patient improved following therapy with meropenem.

### Case 2

**Fig. 4:**
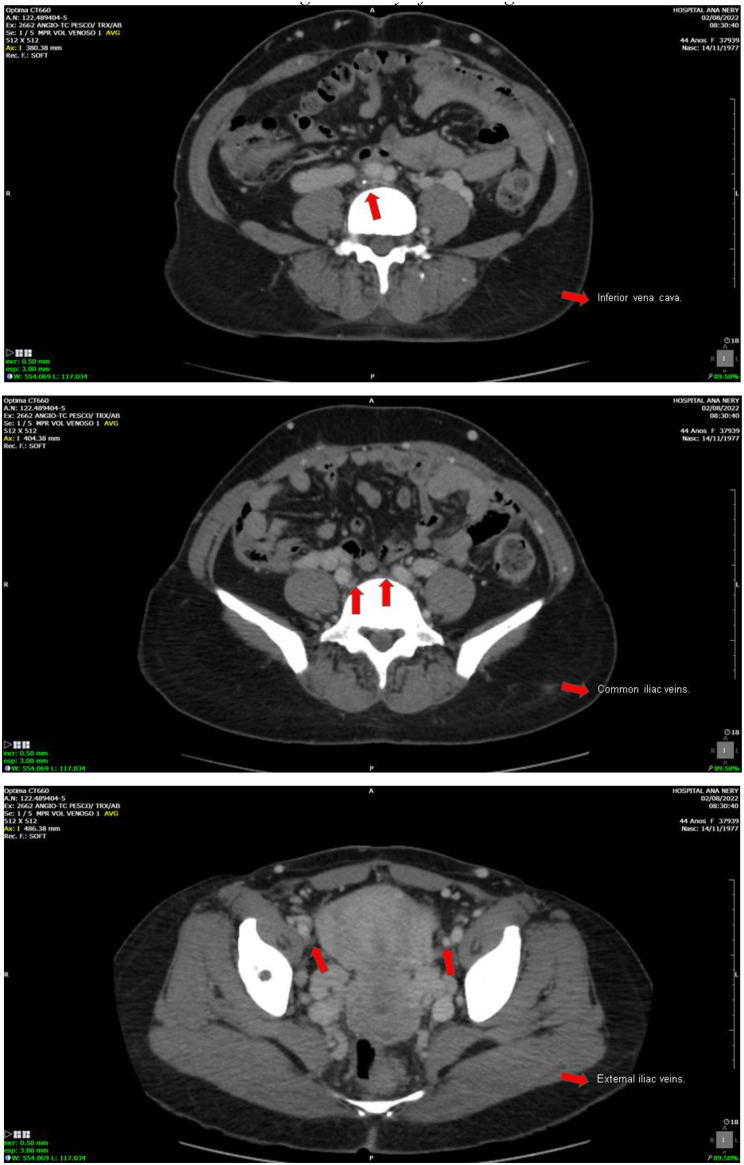
**a-c.** Inferior vena cava with signs of chronic occlusion and luminal calcification. Common iliac veins with signs of chronic occlusion. Right external iliac vein with signs of chronic occlusion and luminal calcification. Left external iliac vein with signs of partial chronic thrombosis and luminal calcification. *Images courtesy of Dr. Thiago Abud Menezes*

A 45-year-old female, who has been undergoing hemodialysis for 180 months, with CKD caused by chronic glomerulonephritis. She presented chronic occlusion and luminal calcification of the inferior vena cava and iliac veins, making conventional venous access unviable (Fig. 4a-c). The CIT in this case was 16 h 15 min. The anastomosis was a termino-lateral anastomosis of the renal vein to the left gonadal vein, and the termino-lateral anastomosis of the renal artery to the infra renal aorta. The surgical technique used for the urinary tract was a uretero-ureteral anastomosis, with the implantation of a double-J ureteral stent.

The immunological risk was high, by the Panel Reactivity Assay (PRA) and by the presence of Donor-Specific Antibodies (DSA), and received Thymoglobulin during induction. For maintenance immunosuppression, PDN, FK, and MPS were used. The graft function was immediate, with an initial creatinine of 5.0 mg/dL, which decreased and stabilized at 0.8 mg/dL after one year. The patient did not require any further surgical interventions and did not present any vascular or infectious complications.

### Case 3

**Fig. 5:**
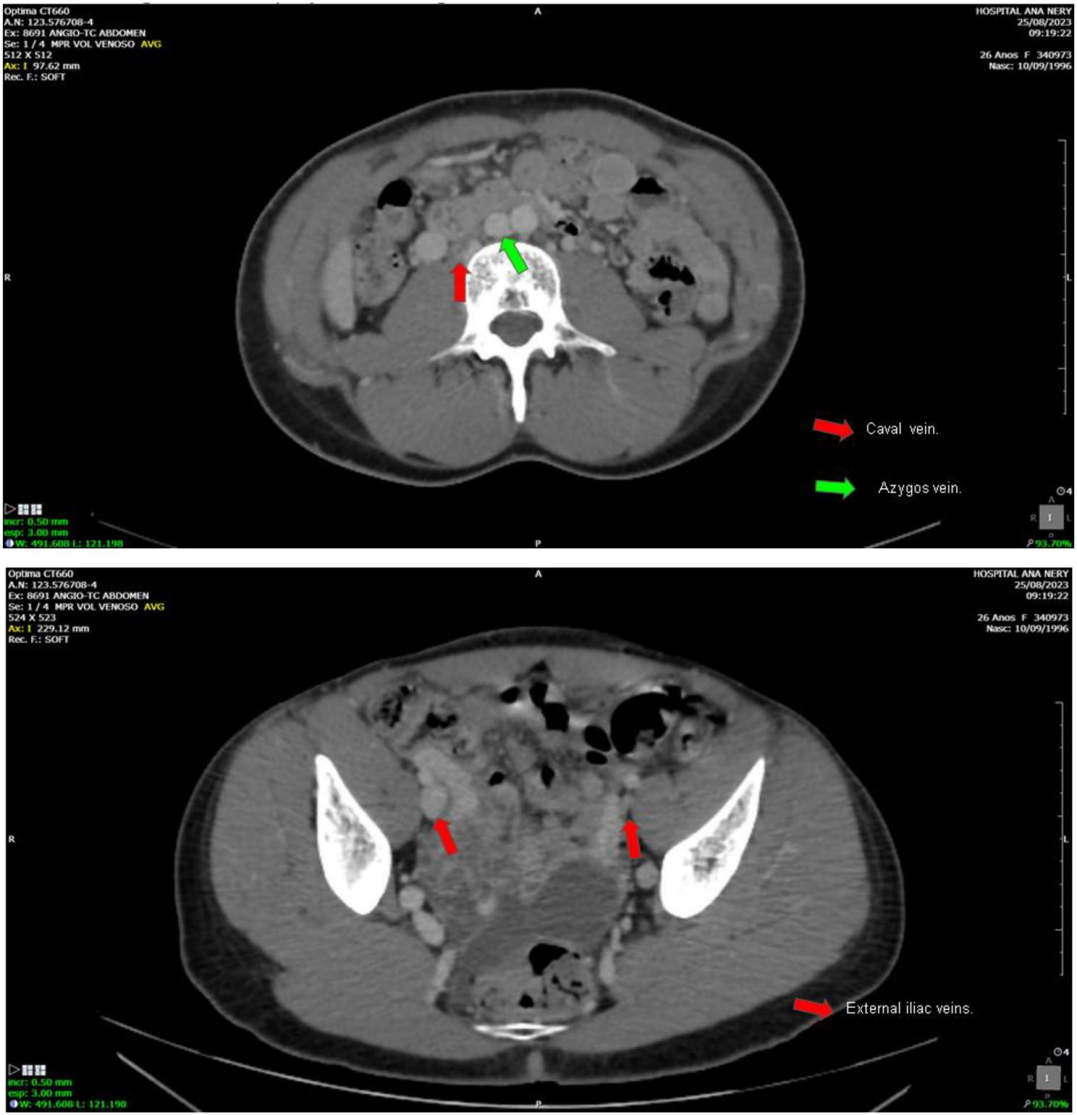
**a-b.** Infra-renal inferior vena cava with signs of chronic occlusion and luminal calcification. Right external iliac vein patent. Left external iliac vein markedly narrowed and showing signs of chronic occlusion/sub-occlusion. *Images courtesy of Dr. Thiago Abud Menezes*

A 27-year-old female, who has been undergoing hemodialysis for 120 months, with an undetermined cause of chronic renal failure. Presenting vascular failure with chronic occlusion and luminal calcification of the infrarenal cava, which made conventional venous access unviable (Fig. 5a-b). The CIT in this case was 23h50min. The anastomosis was a termino-lateral anastomosis of the renal vein to the right gonadal vein, and the termino-lateral anastomosis of the renal artery to the right common iliac artery. The surgical technique used for urinary tract reconstruction was a uretero-ureteral anastomosis, with the placement of a double-J ureteral stent.

The immunological risk was low, and she received thymoglobulin as induction therapy. For maintenance immunosuppression, PDN, FK and azathioprine (AZA) were used. The graft function was immediate, with an initial creatinine of 11.0 mg/dL, which decreased and stabilized at 1.3 mg/dL after one year. The patient did not require any further surgical interventions.

The only complication in the immediate post-transplant period was the diagnosis of histological pyelonephritis associated with antibody-mediated acute rejection. The pyelonephritis was treated with Torgena and Aztreonam, as per the antibiogram, due to the infection caused by Klebsiella pneumoniae producing carbapenemase (KPC). After controlling the infectious condition, the patient underwent plasma exchange sessions as part of the therapy for the rejection.

### Case 4

**Fig. 6:**
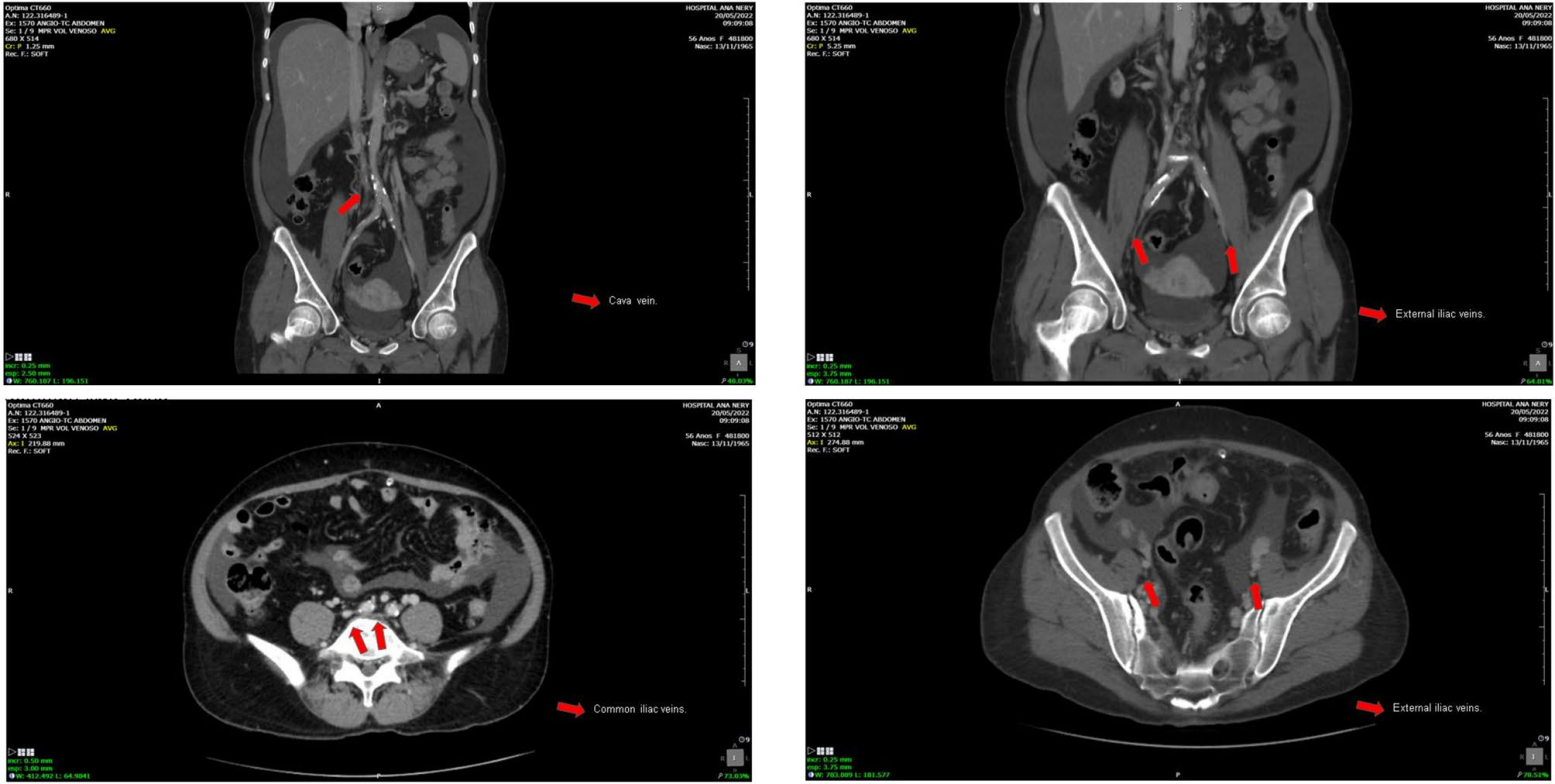
**a-d.** Infrarenal vena cava with signs of chronic occlusion and luminal calcification. Right common iliac vein moderately narrowed. Left common iliac vein with signs of chronic occlusion and luminal calcification. Right external iliac vein with signs of chronic occlusion. Left external iliac vein moderately narrowed with signs of chronic occlusion/subocclusion. *Images courtesy of Dr. Thiago Abud Menezes*

A 27-year-old female, undergoing hemodialysis and peritoneal dialysis for 366 months, with an undetermined cause of chronic renal failure. Vascular failure due to chronic venous occlusion and extensive vascular calcification, making conventional venous anastomosis unfeasible (Fig. 6a-d). The CIT in this case was 26h20min. This particular case involved a differentiated surgical technique (orthotopic). The initial surgical plan for this case was a classic orthotopic approach with venous implantation in the native renal vein and the graft’s renal artery in the aorta, in addition to a left nephrectomy of the native kidney. However, during the surgical procedure, a gonadal vein with good caliber and appearance was identified, and it was decided to implant the graft’s renal vein in it, performing an end-to-side anastomosis rather than an end-to-end anastomosis. Finally, the surgical technique used for urinary tract reconstruction was a uretero-ureteral anastomosis, with the placement of a double-J ureteral stent.

The immunological risk was high, both by the Panel Reactivity Assay (PRA) and received Thymoglobulin during induction. Regarding immunosuppression, PDN, FK, and MPS were used for maintenance therapy. The graft function was immediate, with an initial creatinine of 5.0 mg/dL, which decreased and stabilized at 1.1 mg/dL after three months. The patient did not require any additional surgical interventions. The only complication in the immediate post-transplant period was the diagnosis of pyelonephritis that improved after starting antibiotic therapy; urine culture was negative.

## Discussion

End-stage kidney disease (ESKD) often presents surgical challenges, particularly in patients with iliac vein thrombosis or stenosis, where conventional venous anastomosis is not feasible. In such cases, alternative vascular strategies become essential. Venous options include anastomoses to splanchnic vessels, native renal veins, or the gonadal vein [12], while arterial inflow can be re-established through connections to the aorta or splenic artery [12–14].

Long-term dialysis and repeated vascular access attempts can contribute to iliac vein calcification and thrombosis, significantly increasing the risk of compromised graft perfusion. In this context, the gonadal vein emerges as a viable and underutilized option for venous drainage, as illustrated in our series.

**Fig. 7:**
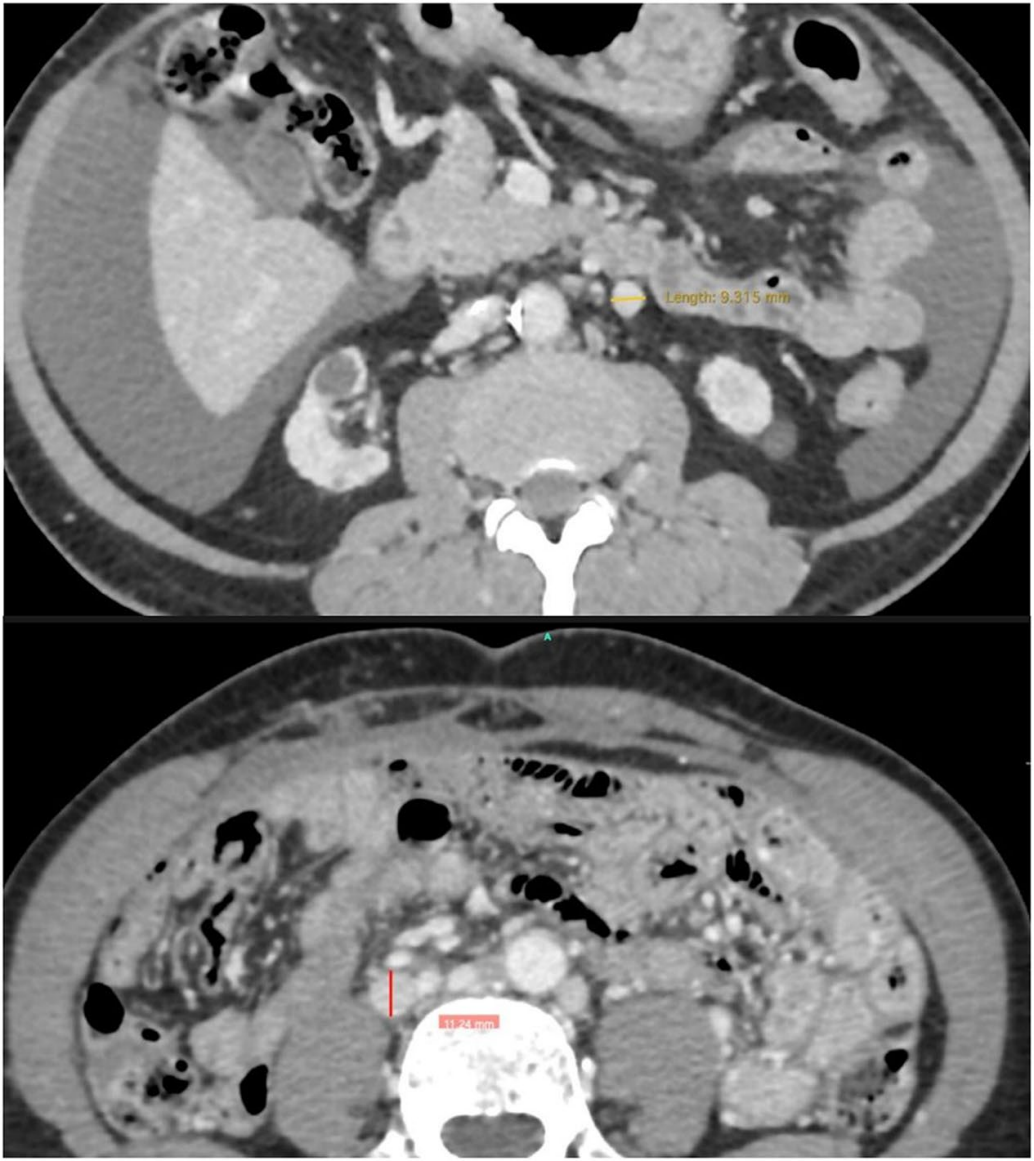
In normal, non-pathological situations, the gonadal vein (ovarian in women) measures up to 5 mm in diameter

 In normal, non-pathological situations, the gonadal vein (ovarian in women) measures up to 5 mm in diameter [15]. In the cases described in this study, we found vicarious gonadal veins as a form of adaptation for chronic occlusions of the infrarenal vena cava. Their diameters ranged from 9 to 11 mm. The choice of these veins as recipients for the venous outflow of the renal graft is not solely due to their increased diameter. Their straight, non-tortuous (varicose) appearance and their connection with the left renal vein (and cava in the right), maintaining cavoatrial outflow, are also taken into consideration in this decision. All surgical planning is performed preoperatively using CT angiography* (*Fig. 7*).*

In all cases described, venous anastomoses were performed using the end-to-side technique. The native gonadal vein was kept in its original position and was not ligated distally. Furthermore, the end-to-side vascular anastomosis technique is easier to proceed and presents a lower risk of stenosis due to technical causes. *Images courtesy of Dr. Rodrigo Mota*

An interesting finding was that the majority of cases were female. Male patients may also be candidates for kidney transplantation with venous anastomosis in the gonadal vein, but there are important anatomical and technical considerations. The male gonadal vein (spermatic vein) is thinner and smaller in caliber than the female gonadal vein (ovarian vein). Therefore, it is a less favorable site for venous anastomosis compared to women. However, in extreme situations, such as extensive thrombosis of the iliocaval system, it may be considered as a salvage option.

When standard anastomosis to the iliac vessels is not viable due to thrombosis or stenosis, vascular alternatives must be considered. For arterial inflow, options such as the common iliac artery or aortic anastomosis may be employed, while the native renal artery serves as a salvage route in selected cases [12, 16]. Regarding venous drainage, the inferior vena cava and native renal vein are commonly used, though in our experience, the gonadal vein has proven to be a useful alternative, particularly for right-sided renal grafts due to its favorable anatomical course. In female patients, the ovarian vein can serve a similar function [12, 17–19].

Compared to a similar report from Ceará, Brazil [12], our cases showed greater surgical variation, including orthotopic transplantation and different arterial sites. While both studies support the feasibility of gonadal vein use, ours included more diverse ureteral techniques and one case of antibody-mediated rejection.

Unlike temporary access routes in patients with poor vascular access, such as transhepatic or popliteal catheterization, gonadal vein anastomosis offers a definitive and technically reproducible solution for venous drainage in select renal transplant recipients with compromised pelvic vasculature. Its use may reduce the need for more invasive or palliative approaches and can provide stable long-term graft function when performed under appropriate anatomical and hemodynamic conditions. Although experience remains limited to case series and reports, the favorable outcomes observed in selected patients support its inclusion among the surgical alternatives for complex transplant scenarios. Nevertheless, careful patient selection, preoperative imaging, and surgical expertise remain essential to optimize results and minimize complications [14, 20].

## Conclusion

The use of the gonadal vein for venous anastomosis in renal transplantation is a viable alternative in patients with thrombosis, stenosis of the iliac vein or infrarenal cava. The cases showed good renal function, few complications, and favorable outcomes. The technique appears safe and effective, but larger studies are needed.

## Data Availability

All data generated or analysed during this study are included in this article. Further inquiries can be directed to the corresponding authors.

## Declarations

### Study location

The present study was carried out in the Nephrology ward and coordinated by the Renal Transplant preceptorship at Hospital Ana Nery, Salvador Bahia, Brazil.
